# Group- and sex-related differences in psychological and pain processing factors in people with and without patellofemoral pain: correlation with clinical outcomes

**DOI:** 10.1186/s12891-023-06513-8

**Published:** 2023-05-19

**Authors:** Ana Flavia Balotari Botta, Júlia de Cássia Pinto da Silva, Helder dos Santos Lopes, Michelle C. Boling, Ronaldo Valdir Briani, Fábio Mícolis de Azevedo

**Affiliations:** 1 Laboratory of Biomechanics and Motor Control (LABCOM), Physical Therapy Department, School of Science and Technology, São Paulo State University (UNESP), Presidente Prudente, Sao Paulo, Brazil; 2 Department of Clinical and Applied Movement Sciences, University of North Florida, Jacksonville, FL USA

**Keywords:** Fear of movement, Catastrophism, Sensitization, Anterior knee pain

## Abstract

**Background:**

People with patellofemoral pain (PFP) exhibit impaired psychological and pain processing factors (i.e., kinesiophobia, pain catastrophizing and pressure pain thresholds [PPTs]). However, it remains unclear whether these factors have different presentations in women and men with PFP, as well as whether their correlation with clinical outcomes differ according to sex. The aims of this study were to: (1) compare psychological and pain processing factors between women and men with and without patellofemoral pain (PFP); (2) investigate their correlation with clinical outcomes in people with PFP.

**Methods:**

This cross-sectional study included 65 women and 38 men with PFP, 30 women and 30 men without PFP. The psychological and pain processing factors were assessed with the Tampa Scale of Kinesiophobia, Pain Catastrophizing Scale, and PPTs of shoulder and patella measured with an algometer. Clinical outcomes assessed were self-reported pain (Visual Analogue Scale), function (Anterior Knee Pain Scale), physical activity level (Baecke's Questionnaire), and physical performance (Single Leg Hop Test). Generalized linear models (GzLM) and effect sizes [Cohen’s *d*] were calculated for group comparisons and Spearman's correlation coefficients were calculated to investigate correlations between outcomes.

**Results:**

Women and men with PFP had higher kinesiophobia (*d* = .82, *p* = .001; *d* = .80, *p* = .003), pain catastrophizing (*d* = .84, p < .001; *d* = 1.27, *p* < .001), and lower patella PPTs (*d* = -.85, *p* = .001; *d* = -.60, *p* = .033) than women and men without PFP, respectively. Women with PFP had lower shoulder and patella PPTs than men with PFP (*d* = -1.24, p < .001; *d* = -.95, *p* < .001), but there were no sex differences in those with PFP for psychological factors (*p* > .05). For women with PFP, kinesiophobia and pain catastrophizing had moderate positive correlations with self-reported pain (rho = .44 and .53, *p* < .001) and moderate negative correlations with function (rho = -.55 and -.58, *p* < .001), respectively. For men with PFP, only pain catastrophizing had moderate positive correlations with self-reported pain (rho = .42, *p* = .009) and moderate negative correlations with function (rho = -.43, *p* = .007).

**Conclusions:**

Psychological and pain processing factors differ between people with and without PFP and between sexes, respectively. Also, correlations between psychological and pain processing factors with clinical outcomes differ among women and men with PFP. These findings should be considered when assessing and managing people with PFP.

**Supplementary Information:**

The online version contains supplementary material available at 10.1186/s12891-023-06513-8.

## Background

Patellofemoral pain (PFP) is one of the most common knee disorders, with an annual prevalence of 23% in the general population and higher incidence in women than in men [1]. The primary symptom of PFP is diffuse anterior knee pain exacerbated by activities such as squatting, running, and walking up and down stairs [2, 3]. PFP negatively impacts physical activity, functional capacity, and social life [4, 5]. Symptoms may persist for up to 18 years after diagnosis [6] and contribute to the development of radiographic signs of patellofemoral osteoarthritis [7].

It is well recognized that impairments in people with PFP are multifactorial, including altered psychological and pain processing factors (i.e., kinesiophobia, pain catastrophizing and pressure pain thresholds [PPTs]) [8, 9]. Kinesiophobia, pain catastrophizing and PPTs have been reported to be impaired in people with PFP [8–10]. They are also reported to be linked with pain and functional outcomes [11, 12]. Yet, the recently published consensus on psychological and pain features in people with PFP still deemed these factors as research priorities and highlighted the need for further investigation [13]. This includes further comparison of psychological and pain processing factors between those with and without PFP, especially within larger cohorts [8, 14].

PFP differs between sexes in many aspects, including epidemiology [1], risk factors [15], biomechanical, and strength impairments [16–18]. Psychological factors and pain processing may also have different presentations in women and men with PFP. In fact, this has already been reported in other pain conditions [19–21], and attributed to biological, psychosocial, and/or cultural differences between sexes [21–23]. In PFP, no study has investigated sex differences regarding psychological factors. In terms of PPTs, a recent systematic review has reported a trend towards women with PFP having lower PPTs than men [14]. Further studies comparing psychological and pain processing factors between women and men with PFP are warranted. Investigating the effect of sex on psychological and pain processing factors even in individuals without pain is also warranted given the scarce literature [24–27].

Psychological and pain processing factors have been reported to be correlated with self-reported pain and function in mixed-sex cohorts of individuals with PFP [12, 28, 29]. It is also warranted to investigate whether correlation between psychological and pain processing factors with clinical outcomes (i.e., self-reported pain, function, physical activity level and physical performance) differ according to sex. Therefore, the aims of this study were to: (1) compare kinesiophobia, pain catastrophizing, and PPTs between women and men with and without PFP; (2) investigate their correlation with self-reported pain, function, physical activity level and physical performance of women and men with PFP. Our hypotheses were psychological and pain processing factors would differ according to sex (i.e., women with and without PFP would present higher pain catastrophizing [20], lower kinesiophobia [19] and lower PPTs [14] than men with and without PFP, respectively) and group (i.e., women and men with PFP would present higher kinesiophobia and pain catastrophizing and lower PPTs than women and men without PFP, respectively). We also hypothesized that psychological and pain processing factors would be significantly correlated with clinical outcomes in women and men with PFP, albeit with increased correlation values in women with PFP.

## Methods

This cross-sectional observational study was reported following STROBE [30] and REPORT-PFP [31].

### Participants

Participants were recruited through social media and advertisements at local universities and gyms in Presidente Prudente (Sao Paulo, Brazil). All interested participants were contacted by phone and scheduled for a face-to-face interview, where a physiotherapist assessed all eligibility criteria. The eligibility criteria for the study were developed based on the latest consensus statement on clinical examination of PFP [32]. To be included in the PFP group, participants had to exhibit: PFP symptoms during activities that load the patellofemoral joint (e.g., squatting, walking up or down stairs, running, jumping); insidious symptoms lasting at least three months; and worst knee pain level of at least 20 mm on a 0–100 mm Visual Analogue Scale (VAS) in the last 30 days [32]. To be included in the control group, participants could not present with any signs and symptoms of PFP. Participants with a diagnosis of any other knee (e.g., meniscal injury, patellar tendon pathology, osteoarthritis) or lower limb disorder or history of knee surgery were not included in either the PFP or control groups.

### Procedures

Initially, demographics such as age, symptoms laterality, and duration of symptoms were verbally obtained from participants. Body mass and height were assessed using a calibrated scale with a stadiometer (WELMY 110; WELMY, Brazil). Participants were then instructed to answer self-administered questionnaires (i.e., Anterior Knee Pain Scale [AKPS], TAMPA Scale for Kinesiophobia [TSK], Pain Catastrophizing Scale [PCS], The Baecke's Habitual Physical Activity Questionnaire). PPTs and physical performance (Single Leg Hop Test [SLHT]) assessments were performed considering the symptomatic limb of those with unilateral PFP or the most symptomatic limb of those with bilateral PFP. For controls, the assessed side was randomly selected.

#### Self-reported pain

Participants were asked to verbally report their worst level of knee pain during the previous month according to a VAS (0–100 mm). The VAS is a reliable and valid tool for assessing self-reported pain of people with PFP [33]. It consists of a 100 mm horizontal line, with 0 representing no pain and 100 the worst pain imaginable. The level of pain was assessed during the eligibility criteria interview.

#### Self-reported function

The AKPS was used to assess self-reported knee function. This tool has been validated for people with PFP, and has been reported to have excellent test–retest reliability [33]. The questionnaire score ranges from 0 (lowest functional capacity) to 100 (highest functional capacity).

#### Kinesiophobia

The TSK was used to assess fear of movement or re-injury due to movement. This tool has been previously validated, and has good test–retest reliability [34]. The score ranges from 17 to 68, with higher scores representing higher kinesiophobia. A cut-off score of 37 can be used to classify individuals as high or low kinesiophobia [35].

#### Pain catastrophizing

The PCS was used to assess the excessively negative orientation towards actual or perceived pain. This is a valid and reliable scale that consists of 13 questions that describe thoughts and feelings that people experience when they feel pain [36]. The scale ranges from 0 to 52, with higher scores representing higher pain catastrophizing. A cut-off score of 24 can be used to classify individuals as high and low pain catastrophizing [28, 37].

#### Physical activity level

The Baecke's Habitual Physical Activity Questionnaire was used to assess physical activity. This is a valid and reliable questionnaire composed of 16 questions distributed in three dimensions: physical activity at work, leisure practices and occupation of free time, and locomotion [38]. Higher scores indicate higher levels of physical activity.

#### Pressure pain thresholds

PPTs are defined as the minimum pressure stimulus perceived as painful and are useful to evaluate hyperalgesia and pain processing alterations [39]*.* PPTs were evaluated using a portable digital pressure algometer (Wagner FPXTM25, USA) with a tip of 1 cm^2^ placed perpendicular to the skin [40, 41]. All measurements were performed by a single assessor trained to exert a pressure of 0.50 kgf/s [40, 41]. Participants were positioned lying supine on an examination table and PPTs were assessed on the center of the patella (local hyperalgesia) and on the shoulder (the lesser tubercle of the humerus) contralateral to the assessed knee (widespread hyperalgesia) (Supplementary Figures S1 and S2) [40, 41]. Participants were instructed to report when the pressure sensation became painful [40, 41]. PPTs were assessed twice at each site with a 30-s interval between assessments, and the mean was used for statistical analysis [40, 41].

#### Single leg hop test

The SLHT was used to assess physical performance. Participants were positioned standing on the tested leg, with the arms crossed on their back and non-stance leg with knee flexed at 90º [18, 42]. Participants were asked to hop forward as far as possible and to land on the same leg while keeping the balance [18, 42]. The distance in centimeters was recorded with a measuring tape considering the heel start and final positions [18, 42]. If participants lost their balance during landing or swung the arms, the trial was not considered valid and was repeated [18, 42]. Three valid repetitions were recorded, and the mean was used for statistical analysis.

### Statistical analysis

Statistical analyses were performed using the Statistical Software for Social Sciences (IBM SPSS Statistics 20) with a level of significance of p < 0.05. A sample size calculation was performed based on results of Sullivan et al. [43], which reported a difference of 9 in PCS with a standard deviation (SD) of 10.4 between women and men without pain. For a two-tailed test, with power of 80% and α = 0.05, the sample size necessary was at least 21 participants per group. All outcomes were tested for normal distribution using the Kolmogorov–Smirnov test. One-way Analysis of Variance (ANOVA) with Bonferroni post-hoc were used to compare demographics between groups. Generalized linear models (GzLM) were used to investigate Group-by-Sex interactions on psychological factors and pain processing. Main effects of group (women and men with PFP vs. women and men without PFP, respectively) and sex (women with and without PFP vs. men with and without PFP, respectively) were reported if there were no significant interactions. As body fat has been correlated with PPTs in people with PFP [44], body mass index (BMI) was included in the model as a covariate. Pairwise comparisons between groups were performed with sequential-Bonferroni post-hoc tests. Mean differences (MD), confidence intervals (CI), and effect sizes (Cohen's *d*) were also calculated. The guidelines for interpreting the Cohen's *d* were small effect (≥ 0.20), moderate effect (≥ 0.50), and large effect (≥ 0.80) [45]. Correlation coefficients among kinesiophobia, pain catastrophizing, PPTs, self-reported pain, function, physical activity level, and physical performance were calculated with Spearman correlation tests for women and men with PFP, separately. The classification of correlation was interpreted as small (< 0.4), moderate (≥ 0.4- < 0.7), and large (≥ 0.7) [45].

## Results

**Fig. 1:**
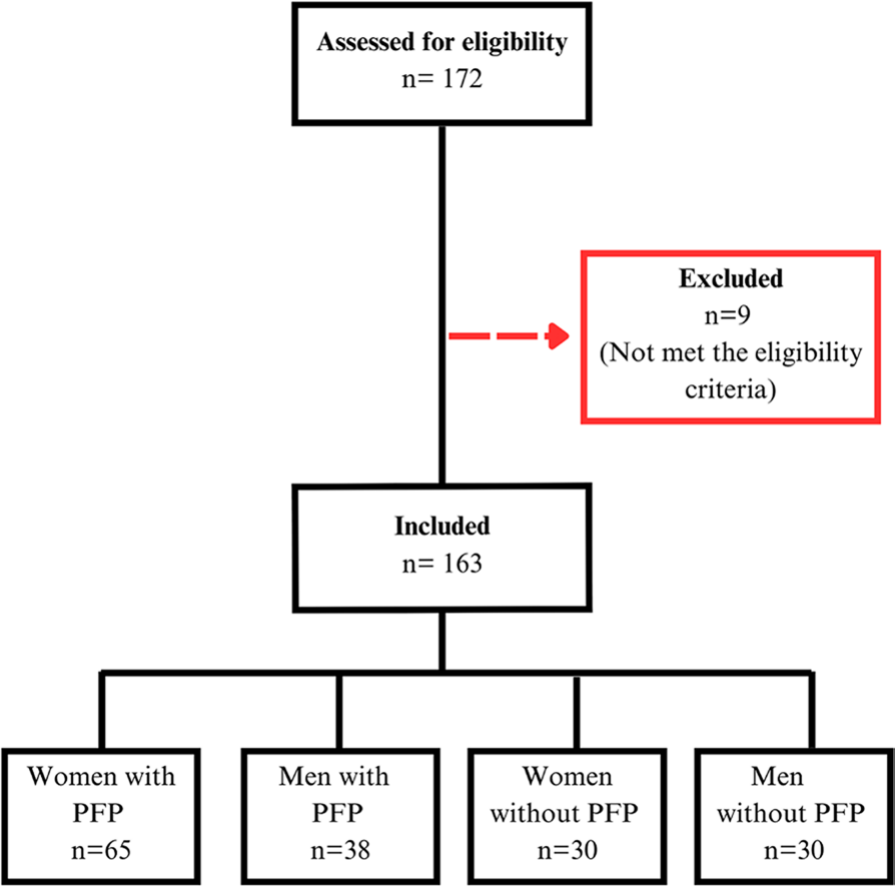
Flow diagram of participants recruitment and selection

One hundred and seventy-two individuals were screened, with 163 meeting the eligibility criteria. They were divided into one of four groups according to sex and the presence of PFP: women with PFP (*n* = 65), men with PFP (*n* = 38), women without PFP (control women) (*n* = 30), men without PFP (control men) (*n* = 30) (Fig. 1).

Mean (SD) for demographics, self-reported measures, PPTs and physical performance are presented in Table 1. Men with PFP were significantly older than men without PFP (*p* = 0.001). Women with PFP had significantly higher height (*p* = 0.041) and body mass (*p* = 0.019) than women without PFP. Women with and without PFP had significant lower height (*p* < 0.001) and body mass (*p* < 0.001 to *p* = 0.005) as compared to men with and without PFP, respectively. However, when considering BMI there were no significant differences between groups. Mean (SD) for self-reported measures, PPTs and physical performance are presented in Table 1.

**Table 1:** Mean (SD) for demographics, self-reported measures, PPTs and physical performance^c^

Variables	Mean (SD)			
***Demographics***	Women without PFP (*n* = 30)	Men without PFP (*n* = 30)	Women with PFP (*n* = 65)	Men with PFP (*n* = 38)
Age (years)	22.33 (2.89)	21.23 (2.22)^b^	23.49 (4.96)	25.08 (4.61)^b^
Height (cm)	160.08 (6.04)^a,b^	176.38 (6.53)^a^	163.79 (5.77)^a,b^	175.75 (6.49)^a^
Body mass (kg)	58.27 (11.52)^a,b^	76.68 (10.36)^a^	67.35 (15.47)^a,b^	76.86 (14.30)^a^
BMI (kg/cm^2^)	22.79 (4.64)	24.68 (2.94)	25.03 (5.12)	24.83 (4.28)
***Self-reported measures***				
TSK	27.23 (5.35)	30.23 (5.53)	33.94 (8.75)	36.21 (7.41)
PCS	3.17 (7.00)	3.37 (6.00)	14.55 (10.83)	18.18 (10.01)
Worst level of pain last month (mm)	NA	NA	55.00 (19.53)	49.34 (19.80)
Symptoms duration (months)	NA	NA	57.91 (52.41)	58.16 (50.13)
Bilateral symptoms (n)	NA	NA	45	18
Self-reported function (AKPS)	98.83 (2.37)	99.07 (2.26)	77.42 (10.97)	80.79 (9.17)
Physical activity level (Baecke)	7.80 (1.20)	8.13 (1.53)	7.90 (1.54)	8.43 (1.54)
***PPTs***				
Shoulder PPTs	2.57 (.726)	4.42 (1.78)	2.75 (.972)	4.03 (1.02)
Patella PPTs	5.14 (1.13)	5.97 (1.17)	4.10 (1.35)	5.23 (1.03)
***Physical performance***				
SLHT (cm)	NA	NA	78.02 (20.54)	111.22 (27.43)

*SD* standard deviation, *PFP* patellofemoral pain, *BMI* body mass index, *TSK* tampa scale for kinesiophobia, *PCS* pain catastrophizing scale, *NA* not assessed, *AKPS* anterior knee pain scale, *Baecke* Baecke's habitual physical activity questionnaire, *PPTs* pressure pain thresholds, *SLHT* single leg hop test. ANOVA comparisons was calculated only for demographics

^a^Represents statistically significant sex differences

^b^Represents statistically significant group differences

^c^Mean (SD) not adjusted by body mass

**Fig. 2:**
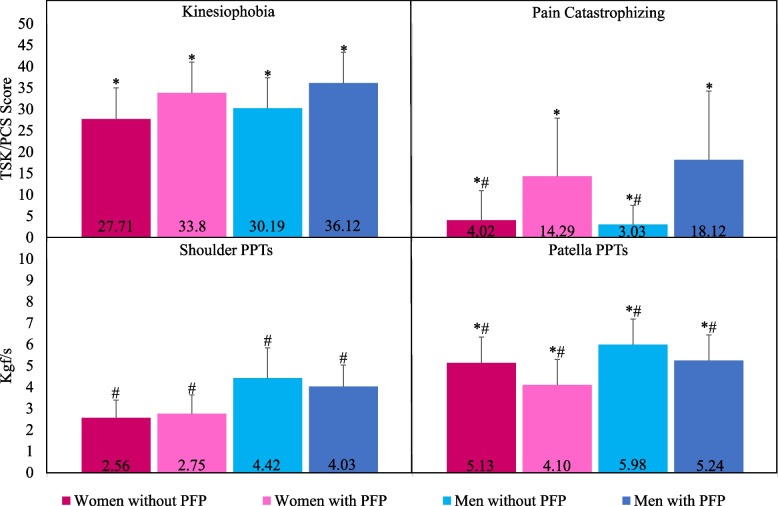
Adjusted mean and between group comparisons for kinesiophobia, pain catastrophizing and PPTs Legend: Abbreviations: PFP: patellofemoral pain; TSK: tampa scale for kinesiophobia; PCS: pain catastrophizing scale; PPTs: pressure pain thresholds. *Group effect. # Sex effect

There was no significant Group-by-Sex interaction (Wald Chi-Square = 0.01; *B* = 0.16; *p* = 0.948) for kinesiophobia. There was also no main effect for sex (Wald Chi-Square = 2.52; *B* = 2.33; *p* = 0.113), but there was a significant main effect for group (Wald Chi-Square = 14.31; *B* = -6.09; *p* < 0.001) (Fig. 2). Post hoc comparisons revealed higher kinesiophobia in women (MD: 6.09; 95% CI: 1.94, 10.24; p = 0.001; *d* = 0.82) and men (MD: 5.93; 95% CI: 1.55, 10.31; *p* = 0.003; *d* = 0.80) with PFP as compared to women and men without PFP, respectively.

There was no significant Group-by-Sex interaction (Wald Chi-Square = 1,82; *B* = -4.82; *p* = 0.177) for pain catastrophizing. There was also no main effect for sex (Wald Chi-Square = 1.52; *B* = 3.83; *p* = 0.218), but there was a significant main effect for group (Wald Chi-Square = 21.99; *B* = -10.27; *p* < 0.001) (Fig. 2). Post hoc comparisons revealed higher pain catastrophizing in women (MD: 10.27; 95% CI: 5.03, 15.51; *p* < 0.001; *d* = 0.84) and men (MD: 15.09; 95% CI: 8.03, 22.14; *p* < 0.001; *d* = 1.27) with PFP as compared to women and men without PFP, respectively.

There was no significant Group-by-Sex interaction (Wald Chi-Square = 2.38; *B* = 0.59; *p* = 0.123) for shoulder PPTs. There was also no main effect for group (Wald Chi-Square = 1.11; *B* = -0.20; *p* = 0.292), but there was a significant main effect for sex (Wald Chi-Square = 29.24; *B* = 1.27; *p* < 0.001) (Fig. 2). Post hoc comparisons revealed lower shoulder PPTs in women with (MD: -1.27; 95% CI: -1.84, -0.71; *p* < 0.001; *d* = -1.24) and without (MD: -1.86; 95% CI: -2.65, -1.07; p < 0.001; *d* = -1.61) PFP as compared to men with and without PFP, respectively.

There was no significant Group-by-Sex interaction for patella PPTs (Wald Chi-Square = 0.54; *B* = -0.29; *p* = 0.485), but there were significant main effects for group (Wald Chi-Square = 14.71; *B* = 1.03; *p* < 0.001) and sex (Wald Chi-Square = 21.68; *B* = 1.14; *p* < 0.001) (Fig. 2). Post hoc comparisons revealed lower patella PPTs in women (MD: -1.03; 95% CI: -1.70, -0.36; *p* = 0.001; *d* = -0.85) and men (MD: -0.74; 95% CI: -1.39, -0.08; *p* = 0.023; *d* = -0.60) with PFP as compared to women and men without PFP, respectively, as well as lower patella PPTs in women with (MD: -1.14; 95% CI: -1.77, -0.51; *p* < 0.001; *d* = -0.95) and without (MD: -0.85; 95% CI: -1.59, -0.10; *p* = 0.020; *d* = -0.68) PFP as compared to men with and without PFP, respectively.

For women with PFP, kinesiophobia and pain catastrophizing had significant moderate positive correlations with self-reported pain (rho = 0.44 to 0.53, *p* < 0.001) and moderate negative correlations with self-reported function (rho = -0.55 to -58, *p* < 0.001). For men with PFP, only pain catastrophizing had a significant moderate positive correlation with self-reported pain (rho = 0.42, *p* = 0.009) and moderate negative correlation with self-reported function (rho = -0.43, *p* = 0.007). Kinesiophobia had no significant correlations with self-reported pain and function in men with PFP (*p* > 0.05). Shoulder PPTs had a significant small positive correlation only with self-reported pain in men with PFP (rho = 0.34, *p* = 0.035). Patella PPTs had significant small positive correlations with self-reported function (rho = 0.33, *p* = 0.008) and physical performance (rho = 0.34, *p* = 0.005) in women with PFP. Physical activity level had no significant correlations with any psychological or pain processing factors (*p* > 0.05). Scatterplots are depicted in Supplementary material.

## Discussion

This study aimed to investigate if psychological and pain processing factors have different presentations in women and men with and without PFP and if their correlations with clinical outcomes also differ according to sex. Overall, our results indicate group-related (PFP vs. control) differences in psychological and local pain processing factors, while sex-related differences were present only in pain processing factors. In individuals with PFP, sex-related differences in correlations with clinical outcomes were also observed.

Our findings are in line with previous studies [8, 46] indicating higher levels of kinesiophobia and pain catastrophizing in people with PFP. However, our hypotheses that there would be sex differences in kinesiophobia and pain catastrophizing were not confirmed. It is suggested that kinesiophobia and pain catastrophizing develop as a maladaptive response to negative pain experiences, which lead people with chronic pain to be excessively vigilant and to avoid stimuli perceived to be painful [21, 47]. Although sex has been previously reported to play a role in kinesiophobia and pain catastrophizing [19–21], PFP symptoms seems to have a greater influence than sex, which may explain our findings. As kinesiophobia and pain catastrophizing are considered important in treatment planning, clinical examination, and prognostication [13], they should be assessed in both women and men with PFP.

The correlation between pain catastrophizing and self-reported pain and function were slightly higher in women than in men with PFP, while kinesiophobia correlated significantly with these outcomes only in women with PFP. Correlations of kinesiophobia and pain catastrophizing with self-reported pain and function have already been reported in mixed-sex cohorts predominantly composed of women (72–89.4%) [12, 28, 29]. Our study is the first to demonstrate that, although elevated when compared to controls, kinesiophobia is not correlated with pain and function in men with PFP. As such, higher levels of kinesiophobia are reported by men with PFP regardless of pain intensity. The use of the TSK questionnaire might assist clinicians to identify patients whose fear of movement may negatively impact their rehabilitation, especially as kinesiophobia does not seem to be directly correlated with pain levels in men with PFP. It should also be noted that even though people with PFP in our study had significantly higher psychological factors than controls, mean values did not exceed the cut-off scores to be classified with high levels of kinesiophobia and pain catastrophizing [28, 35, 37]. It is possible that in populations presenting values above cut-off scores, correlations with clinical outcomes may differ.

Interventions targeting kinesiophobia and pain catastrophizing have been previously proposed [48–52]. The majority of them consist of cognitive interventions focusing on education about pain mechanisms, psychological beliefs, coping with symptoms, load management and gradual exposure to physical activity [48–50]. Even though psychological factors were not classified as high in our cohort of people with PFP, they are still elevated as compared to controls. Thus, interventions targeting psychological factors may be advised for women and men with PFP. Given high levels of kinesiophobia seem to be reported by men with PFP regardless of pain intensity, interventions targeting kinesiophobia may be especially important for this subgroup of people with PFP.

The fear-avoidance model’s assumption that worst psychological factors are correlated with worst physical activity levels and physical performance [47] is not supported by our findings. Other studies also did not find correlations between psychological factors, physical activity levels, and physical performance in people with PFP [28, 46]. Although people with PFP have elevated psychological factors, they would still be able to remain physically active and have similar performance in functional tasks as asymptomatic people [4, 28]. However, those who experience fear of pain may adapt their movement pattern in order to minimize subsequent painful stimuli [53]. This is in line with a study that reported significant correlations between fear avoidance belief and single leg squat hip adduction, step-down knee abduction, jogging knee abduction, and jogging hip adduction in women with PFP [54]. Similarly, another study reported significant correlations between kinesiophobia and peak knee flexion during stair descent in women with PFP [55]. Future studies are warranted to investigate whether the correlation between kinesiophobia and movement pattern also applies to men with PFP.

In accordance with a recent meta-analysis [9], women and men with PFP presented with lower patella PPT as compared to women and men without PFP, respectively. Our results also showed lower patella PPTs in women with and without PFP as compared to men with and without PFP. There is evidence that women are more sensitive to pressure pain than men [23, 27]. Women with PFP had the lowest patella PPTs, which may be the result of a cumulative effect of sex and painful condition. Quantitative sensory testing was determined a research priority for treatment prediction, pathophysiology, and prognosis in the recently published consensus on pain and psychological features [13]. Our findings implies that future research should investigate women and men with PFP separately given the possible influence of sex on the findings.

In contrast to previous findings [9], no differences in shoulder PPTs were found between people with PFP and controls, only a sex effect was observed. This finding is in agreement with a recent study that reported lower PPTs in women with knee pain as compared to men with knee pain, but no differences in those with and without radiographic knee osteoarthritis [56]. Two additional studies also reported no manifestations of widespread hyperalgesia in a PFP population, endorsing that this is not an incontestable finding in people with PFP [57, 58]. A recent meta-regression indicated positive correlations between age and PPTs in people with PFP [14], which may explain the conflicting findings. Evidence suggests that younger people are more likely to experience pressure pain sensitivity [14]. Further investigations are warranted to determine whether the findings of pressure pain sensitivity are indeed age dependent.

Local and widespread PPTs had only small correlations with self-reported pain, function, and physical performance, as well as no correlations with physical activity level and psychological factors. A recent study also did not report correlations of kinesiophobia and pain catastrophizing with quantitative sensory tests [28]. Instead, local and widespread hyperalgesia have been recently reported to be correlated with body fat and skeletal muscle mass of people with PFP, although the amount of variance explained was generally low [44]. Therefore, these findings are in line with the recently published consensus that regarded quantitative sensory testing as not clinically important [13].

Some limitations of this study must be acknowledged. This study has a cross-sectional design, thus no causality can be inferred. Only young adults were included in our study, which may limit the generalizability of our findings to adolescents and older individuals. We have only assessed kinesiophobia and pain catastrophizing as they are deemed the most investigated and clinically important psychological factors in people with PFP [13]. Further investigation addressing other factors such as anxiety, depression, and self-efficacy are warranted. The same applies to assessing only PPTs, which have the highest research priority in quantitative sensory testing [13]. The investigation of temporal summation and conditioned pain modulation may provide additional information on pain processing aspects [59]. Lastly, although physical performance was assessed with the SLHT, we did not investigate the movement pattern during the test (i.e., kinematics, kinetics).

## Conclusions

Psychological factors did not differ according to sex in people with PFP, while shoulder and patella PPTs were lower in women than men with PFP. Women and men with PFP had impaired psychological factors and local pain processing as compared to controls. Kinesiophobia correlated with clinical outcomes only in women with PFP. Sex differences in psychological and pain processing factors should be considered when assessing and managing people with PFP.

## Supplementary Information

**Additional file 1.** 

## Data Availability

The datasets used and/or analysed during the current study are available from the corresponding author on reasonable request.
