# Supplementary material for: Group- and sex-related differences in psychological and pain processing factors in people with and without patellofemoral pain: correlation with clinical outcomes
Source: BMC Musculoskelet Disord. 2023 May 19;24:397. doi: 10.1186/s12891-023-06513-8 (PMC10197856; doi:10.1186/s12891-023-06513-8)
Supplement: Supplementary file 1 — Additional file 1. [file 12891_2023_6513_MOESM1_ESM.pdf]

Supplementary Material

Figure S1 – Positioning for patella pressure pain thresholds assessment.

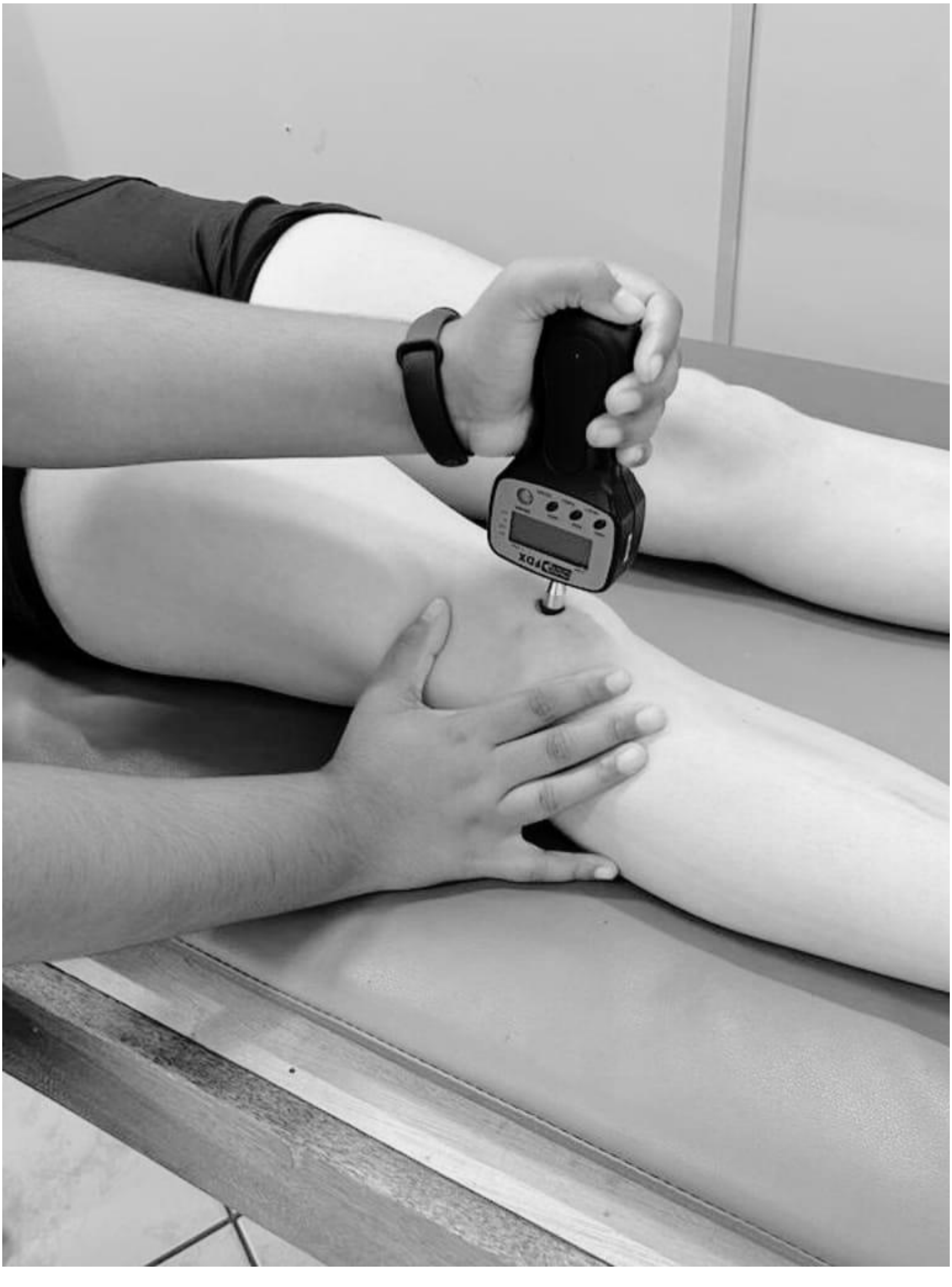

Figure S2 – Positioning for shoulder pressure pain thresholds assessment.

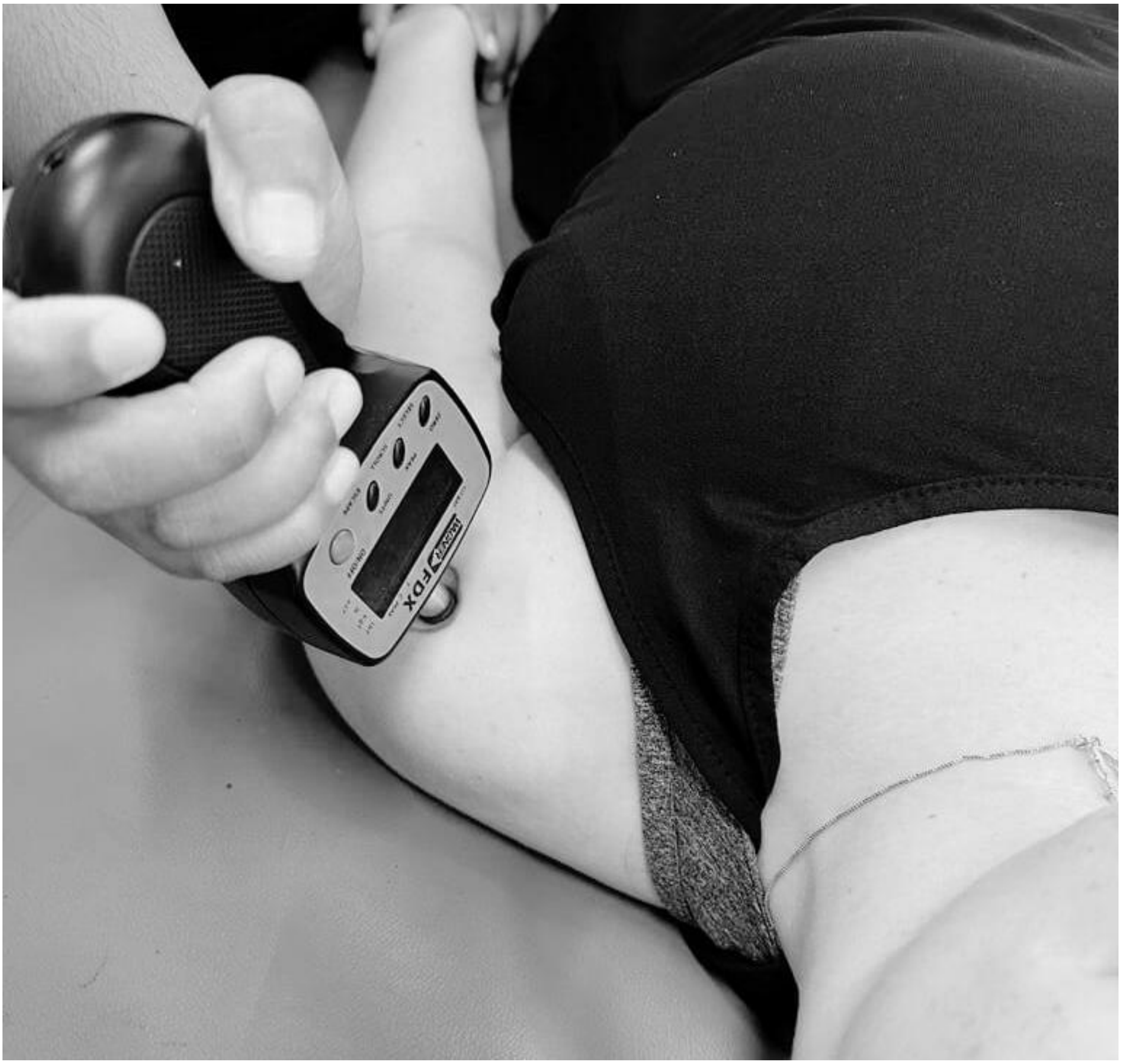

Figure S3a – Correlation of TSK with shoulder and patella PPTs (kgf/s), worst level of pain last month (mm), AKPS; Baecke and SLHT (cm) for women with PFP.

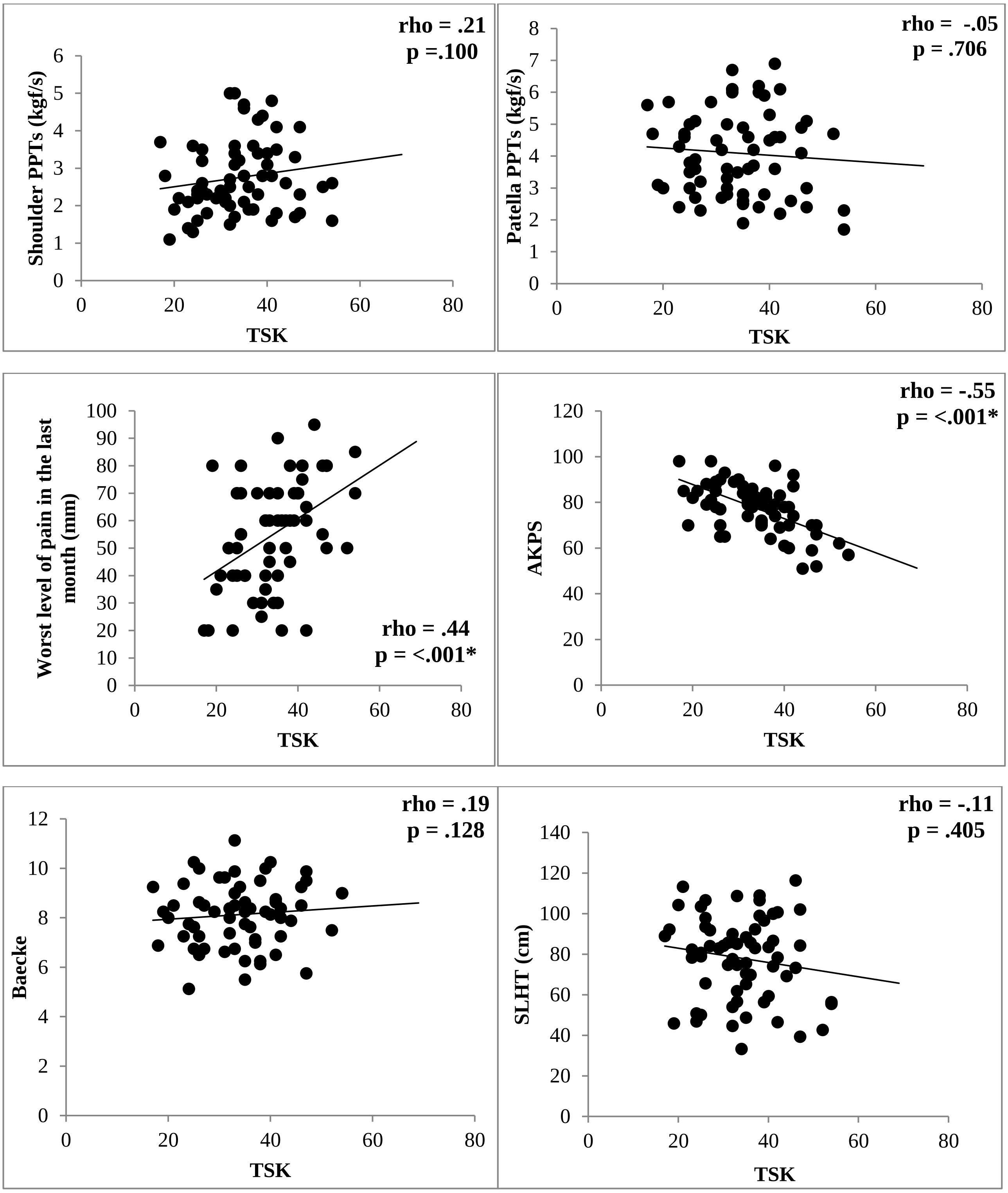

Abbreviations: TSK: tampa scale for kinesiophobia; PPTs: pressure pain thresholds; AKPS: anterior knee pain scale; Baecke: Baecke's Habitual Physical Activity Questionnaire. SLHT: single leg hop test. PFP: Patellofemoral pain.

Figure S3b – Correlation of PCS with shoulder and patella PPTs (kgf/s), worst level of pain last month (mm), AKPS; Baecke and SLHT (cm) for women with PFP

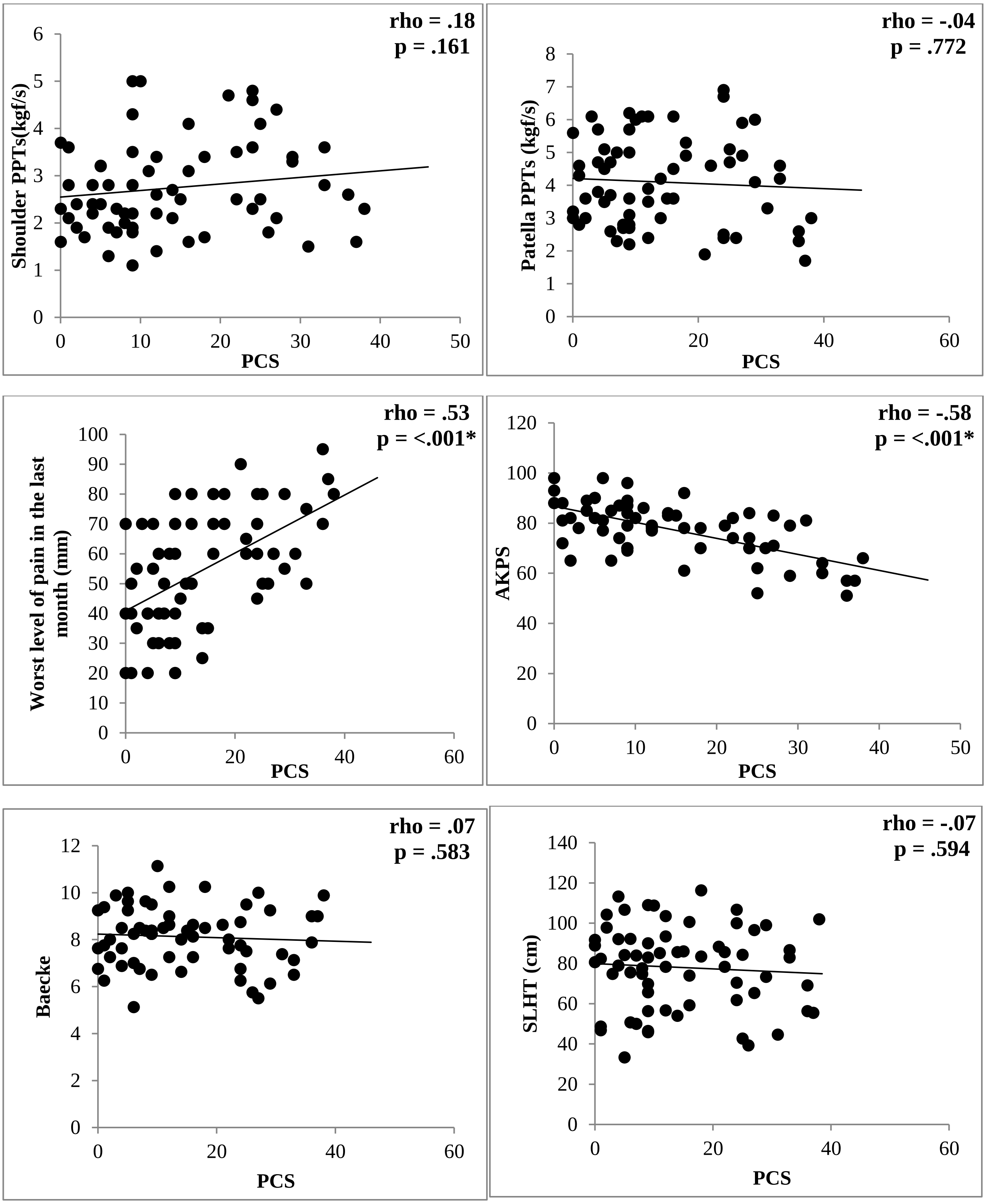

Abbreviations: PCS: pain catastrophizing scale; PPTs: pressure pain thresholds; AKPS: anterior knee pain scale; Baecke: Baecke's Habitual Physical Activity Questionnaire; SLHT: single leg hop test. PFP: Patellofemoral pain. *Represents statistical significance.

Figure S3c – Correlation of shoulder PPTs (kgf/s) with worst level of pain last month (mm), AKPS; Baecke and SLHT (cm) for women with PFP.

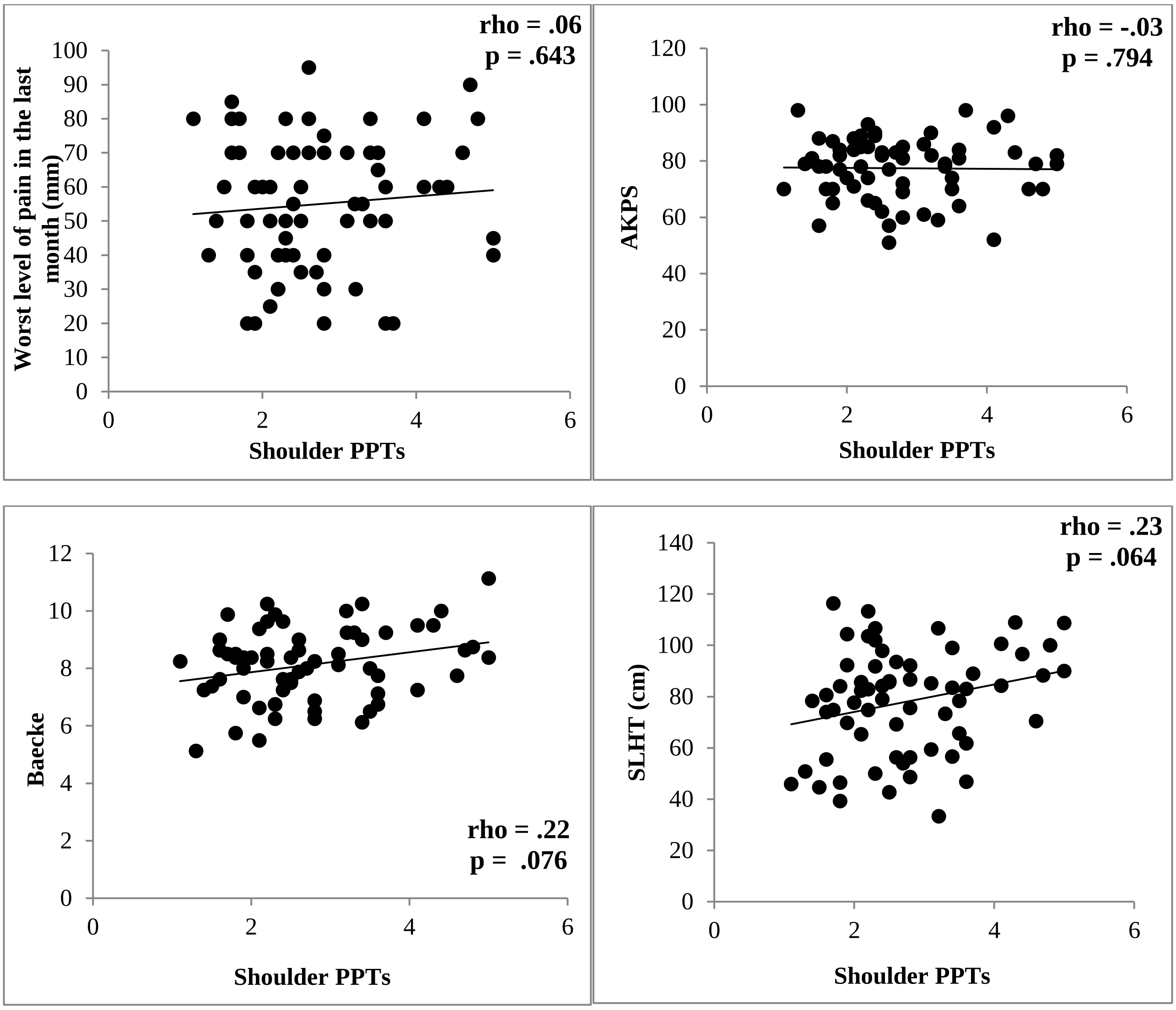

Abbreviations: PPTs: pressure pain thresholds; AKPS: anterior knee pain scale; Baecke: Baecke's Habitual Physical Activity Questionnaire; SLHT: single leg hop test. PFP: Patellofemoral pain.

Figure S3d – Correlation of patella PPTs (kgf/s) with worst level of pain last month (mm), AKPS; Baecke and SLHT for women with PFP.

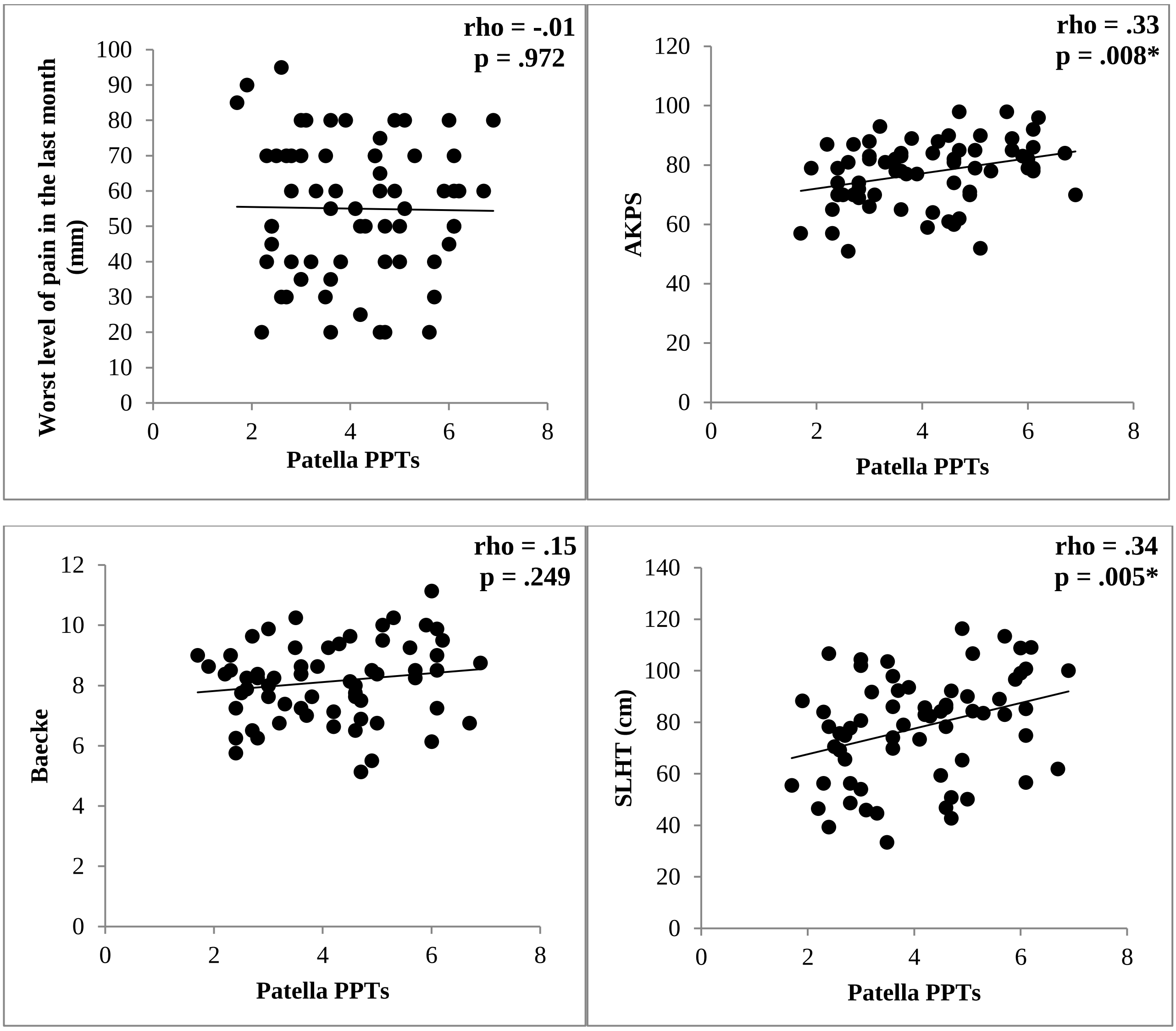

Abbreviations: PPTs: pressure pain thresholds; AKPS: anterior knee pain scale; Baecke: Baecke's Habitual Physical Activity Questionnaire; SLHT: single leg hop test. PFP: Patellofemoral pain.

Figure S4a – Correlation of TSK with shoulder and patella PPTs (kgf/s), worst level of pain last month (mm), AKPS; Baecke and SLHT (cm) for men with PFP.

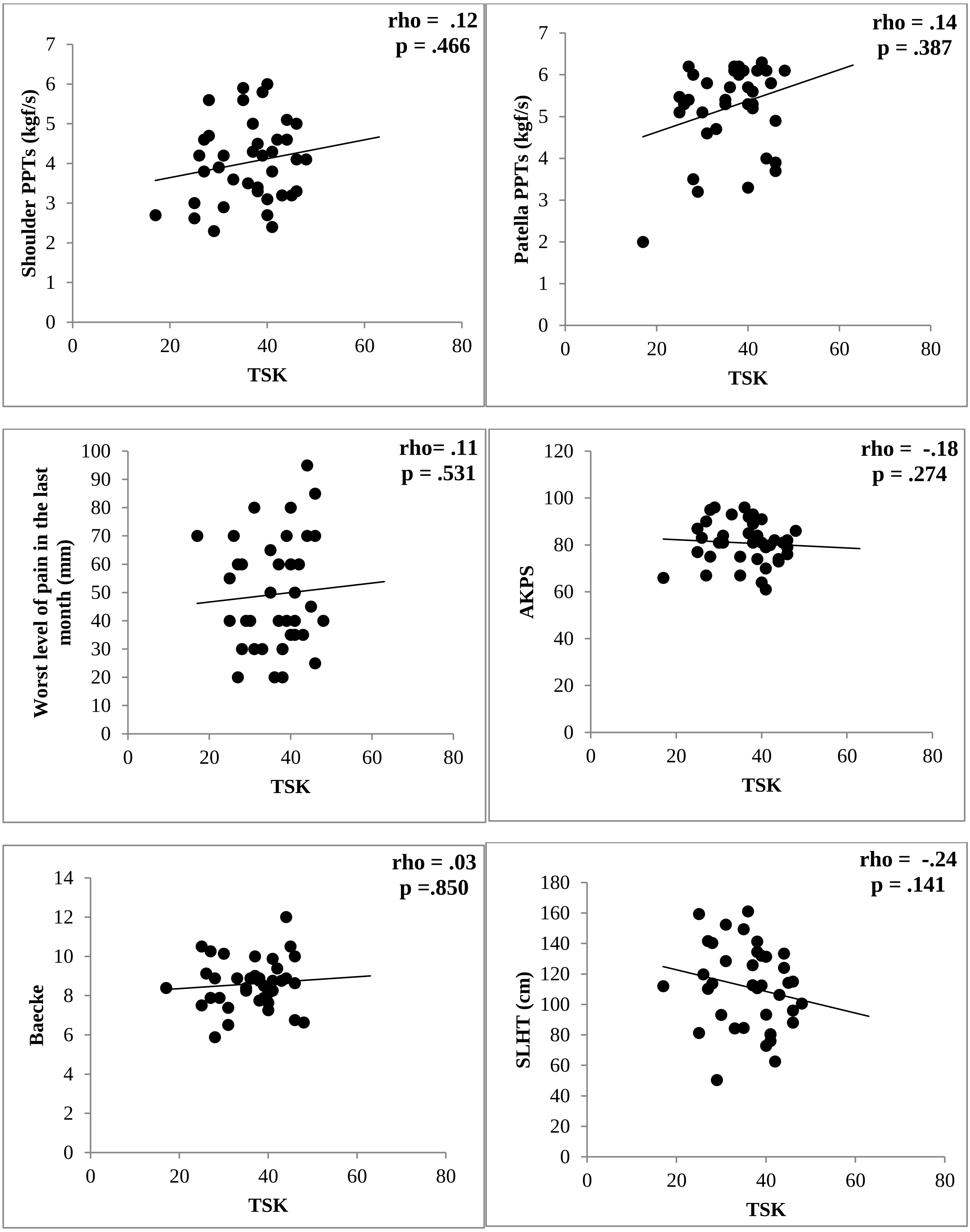

Abbreviations: TSK: tampa scale for kinesiophobia; PPTs: pressure pain thresholds; AKPS: anterior knee pain scale; Baecke: Baecke's Habitual Physical Activity Questionnaire. SLHT: single leg hop test. PFP: Patellofemoral pain.

Figure S4b – Correlation of PCS with shoulder and patella PPTs (kgf/s), worst level of pain last month (mm), AKPS; Baecke and SLHT (cm) for men with PFP.

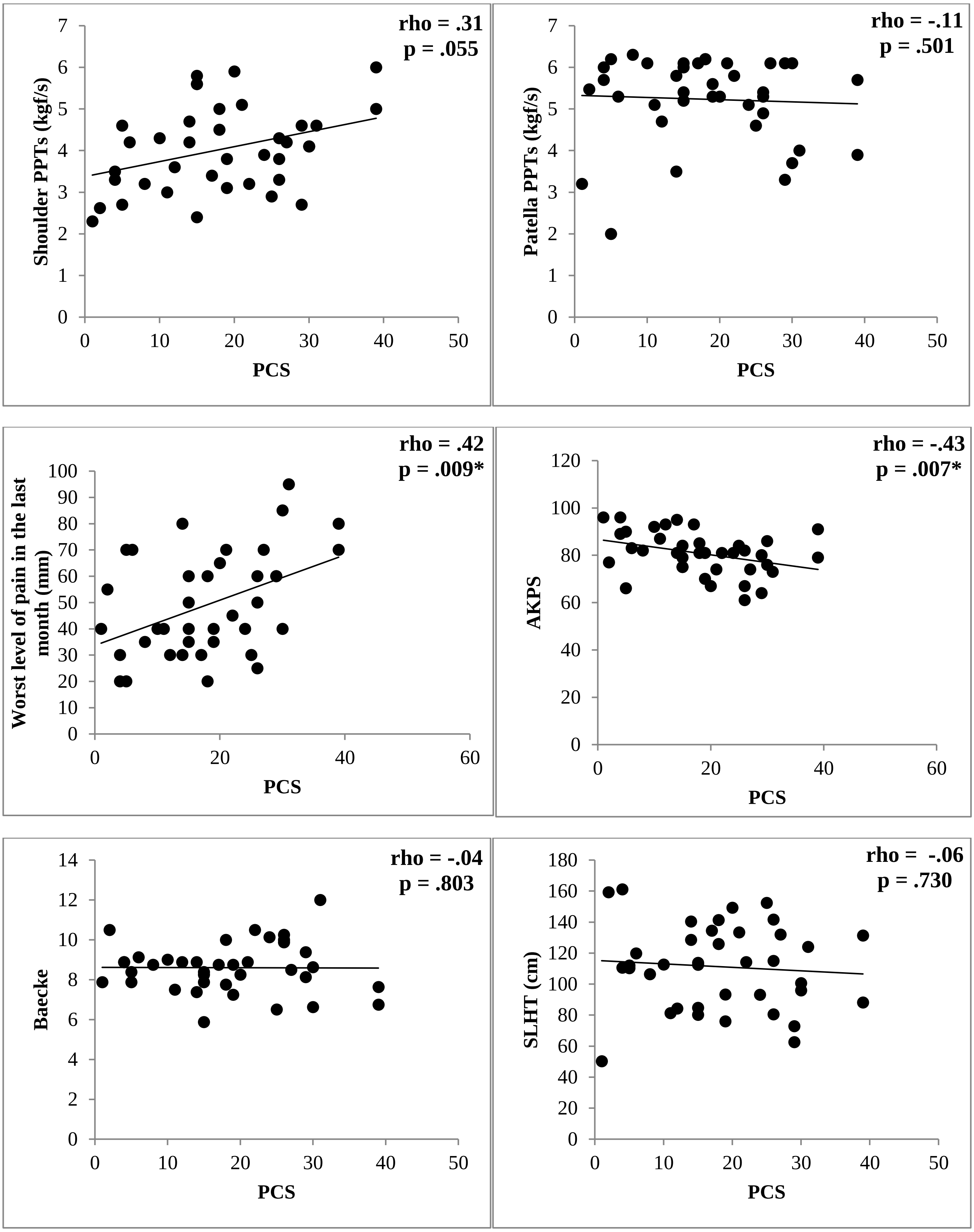

Abbreviations: PCS: pain catastrophizing scale; PPTs: pressure pain thresholds; AKPS: anterior knee pain scale; Baecke: Baecke's Habitual Physical Activity Questionnaire; SLHT: single leg hop test. PFP: Patellofemoral pain. *Represents statistical significance.

Figure S4c – Correlation of shoulder PPTs (kgf/s) with worst level of pain last month (mm), AKPS; Baecke and SLHT (cm) for men with PFP.

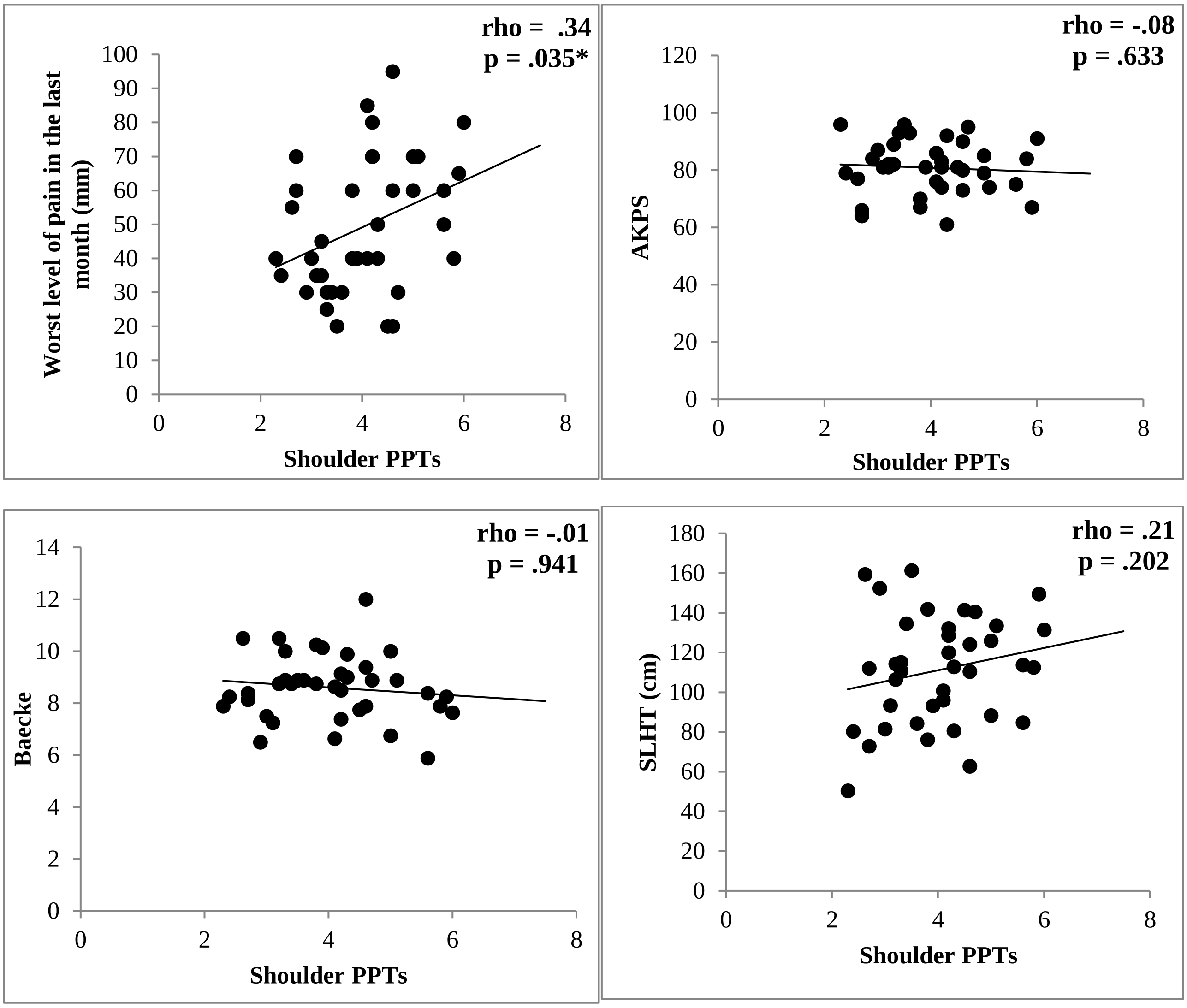

Abbreviations: PPTs: pressure pain thresholds; AKPS: anterior knee pain scale; Baecke: Baecke's Habitual Physical Activity Questionnaire; SLHT: single leg hop test. PFP: Patellofemoral pain. *Represents statistical significance.

Figure S4d – Correlation of patella PPTs (kgf/s) with worst level of pain last month (mm), AKPS; Baecke and SLHT (cm) for men with PFP.

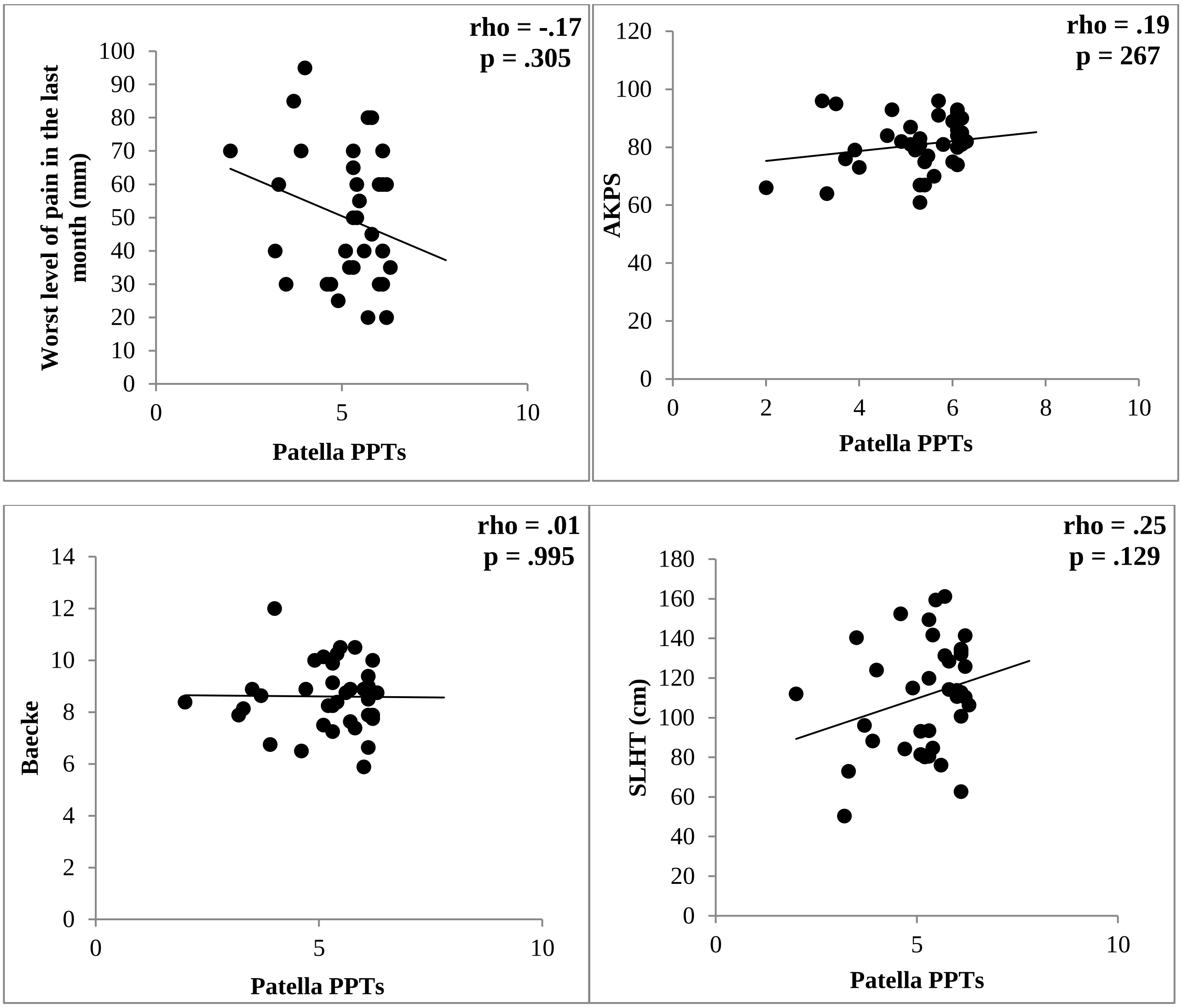

Abbreviations: PPTs: pressure pain thresholds; AKPS: anterior knee pain scale; Baecke: Baecke's Habitual Physical Activity Questionnaire; SLHT: single leg hop test. PFP: Patellofemoral pain.
